# Hydrogel Crosslinked with Nanoparticles for Prevention of Surgical Hemorrhage and Recurrence of Hepatocellular Carcinoma

**DOI:** 10.1002/advs.202305508

**Published:** 2023-12-25

**Authors:** Jia‐Qi Zhu, Han Wu, Xu Li, Min‐Yu Li, Zhen‐Li Li, Xin‐Fei Xu, Li‐Hui Gu, Dong‐Xu Yin, Feng Shen, Dong‐Sheng Huang, Tian Yang

**Affiliations:** ^1^ Department of Hepatobiliary Surgery Eastern Hepatobiliary Surgery Hospital Second Military Medical University (Naval Medical University) Shanghai 200438 China; ^2^ College of Biotechnology and Bioengineering Zhejiang University of Technology Hangzhou Zhejiang 310014 China; ^3^ Department of Colorectal Surgery The First Affiliated Hospital of Naval Medical University Shanghai 200433 China; ^4^ Department of Special Care Unit The First Affiliated Hospital of Naval Medical University Shanghai 200433 China; ^5^ School of Clinical Medicine Hangzhou Medical College Hangzhou Zhejiang 310014 China; ^6^ The Key Laboratory of Tumor Molecular Diagnosis and Individualized Medicine of Zhejiang Province Zhejiang Provincial People's Hospital (People's Hospital of Hangzhou Medical College) Hangzhou Zhejiang 310014 China

**Keywords:** adhesive hydrogels, block copolymer, hepatocellular carcinoma, immunotherapy, tumor recurrence

## Abstract

Hepatocellular carcinoma (HCC) is acknowledged as an immunosuppressive neoplasm, whereby the inactive microenvironment facilitates immune tolerance and evasion of HCC. Post‐surgical resected liver cancer exhibits a proclivity for relapse, rendering prevention of recurrence challenging as it may transpire at any point subsequent to surgery. Among the various anti‐recurrence interventions, the primary clinical approach involving the administration of regimens atezolizumab and bevacizumab (A+T) is deemed the most efficacious in reversing the tumor microenvironment, albeit still lacking in complete satisfaction. Therefore, the objective is to utilize a recently developed block copolymer as a protective carrier for two specific monoclonal antibody drugs. Subsequently, a modified hemostatic hydrogel will be synthesized for application during hepatic surgery. The immunotherapy impact of this approach is significantly prolonged and intensified due to the combined hemostasis properties and controlled release of the constituents within the synthesized nanocomposite hydrogel. Furthermore, these nanocomposite hydrogels exhibit remarkable efficacy in preventing postoperative wound bleeding and substantially enhancing the safety of liver cancer resection. This research on the anti‐recurrence hydrogel system presents a novel therapeutic approach for addressing local recurrence of liver cancer, potentially offering a substantial contribution to the field of surgical treatment for liver cancer in the future.

## Graphical abstract

Among the various anti‐recurrence interventions, the primary clinical approach involving the administration of regimens atezolizumab and bevacizumab is deemed the most efficacious in reversing the tumor microenvironment, albeit still lacking in complete satisfaction. This research on the anti‐recurrence hydrogel system presents a novel therapeutic approach for addressing local recurrence of liver cancer, potentially offering a substantial contribution to the field of surgical treatment for liver cancer in the future.

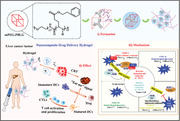

## 1 Introduction

The global incidence of hepatocellular carcinoma (HCC) is increasing.^[^ ^1^ ^]^ Contemporary medical advancements have led to a diverse range of treatment options for liver cancer, predominantly encompassing surgical interventions, liver transplantation, ablation techniques, interventional therapy, radiation therapy, chemotherapy, targeted therapy, and immunotherapy.^[^ ^2^, ^3^ ^]^ Although the occurrence of HCC after surgery is a significant issue, the use of nanotechnology shows immense potential in enhancing the effectiveness of immunotherapy for individuals suffering from advanced cancer.^[^ ^4^, ^5^ ^]^ Unlike traditional cancer immunotherapies, intricately engineered nanomaterials can induce targeted tumoricidal effects, enhancing the immune cell's ability to reach crucial metastatic locations like the bone, lung, and lymph nodes.^[^ ^6^, ^7^, ^8^, ^9^ ^]^ This phenomenon enhances the display of antigens and results in enduring immune reactions. In accordance with global standards, the recommended therapeutic approach for advanced HCC in previously untreated individuals, yielding superior overall and progression‐free survival rates, involves the administration of atezolizumab and bevacizumab in combination, contingent upon meeting the eligibility criteria established in the pivotal clinical trial.^[^ ^10^ ^]^ Nevertheless, there exist distinct patient subgroups with specific pre‐existing ailments that may render them unsuitable candidates for this innovative treatment due to either safety apprehensions or potential lack of efficacy.^[^ ^11^ ^]^

The utilization of immunotherapy exhibits promising prospects in augmenting the survival rates of patients diagnosed with HCC.^[^ ^12^ ^]^ By focusing on the immune system's capacity to identify and eradicate cancerous cells, it operates effectively. HCC has a unique tumor microenvironment that is immunosuppressive, allowing the tumor to avoid detection by the immune system.^[^ ^13^ ^]^ This immunosuppressive setting is influenced by the existence of specific immune cells, referred to as tumor‐infiltrating lymphocytes, which encompass CD8‐positive cytotoxic T cells, as well as the presence of immune checkpoint molecules like programmed death‐ligand 1 (PD‐L1).^[^ ^14^ ^]^ In order to address this issue, the utilization of two monoclonal antibody drugs, namely atezolizumab and bevacizumab, has been incorporated into the treatment of HCC. Atezolizumab, functioning as a PD‐L1 inhibitor, impedes the interaction between PD‐L1 and programmed cell death protein 1 (PD‐1), thereby reinstating the immune response against malignant cells. Conversely, bevacizumab acts as a vascular endothelial growth factor (VEGF) inhibitor, effectively suppressing tumor angiogenesis, a pivotal process that facilitates the provision of nutrients and oxygen to the tumor, thereby promoting its dissemination. In the clinical trials for advanced HCC, the use of atezolizumab and bevacizumab has shown encouraging results, significantly improving both overall survival and progression‐free survival compared to the conventional treatment method.^[^ ^15^ ^]^

In order to surpass these constraints, scientists have investigated innovative methods of transporting drugs that specifically target the tumor location, reducing overall exposure and improving the effectiveness of treatment. Hydrogel is a specific model of cancer treatment with potential adhesive properties for numerous biomedical applications.^[^ ^16^ ^]^ It possess a substantial amount of water and are compatible with living organisms, find diverse uses in the realms of biomedicine, including cancer, ^[^ ^17^ ^]^ diabetes,^[^ ^18^ ^]^ and cardiovascular disease.^[^ ^19^ ^]^ To address the limitations of conventional gels regarding the rate of drug loading and gradual release, our objective was to investigate if the application of nanocomposite hydrogels loaded with immune agonists at the tumor resection site, before closing the surgical wound, could effectively revert the immune condition of the main tumor and inhibit it through postoperative immunotherapy. With the inspire of polymers have a lot of advantages including good biocompatibility and low costs. Compared with traditional hydrogel, we designed a nanocomposite hydrogel that could sustainably release drugs within a long time. Amphiphilic copolymers comprising poly(ethylene glycol) (PEG) as the hydrophilic block and copolymers of lactide, glycolide, and ε‐caprolactone have been widely investigated for preparation of biodegradable and drug loaded hydrogels.^[^ ^20^, ^21^ ^]^ The rate of tumor recurrence is expected to decrease with timely local immune enhancement therapy after the surgical procedure. Adding adhesive characteristics to hydrogels can greatly enhance or broaden their potential uses. A range of durable adhesive hydrogels has been successfully created in previous research through the formation of amide bonds.^[^ ^22^ ^]^ These nanocomposite hydrogels have been utilized in diverse applications such as tissue adhesives, wound dressings, and tissue repairs.^[^ ^23^ ^]^

**Scheme 1:**
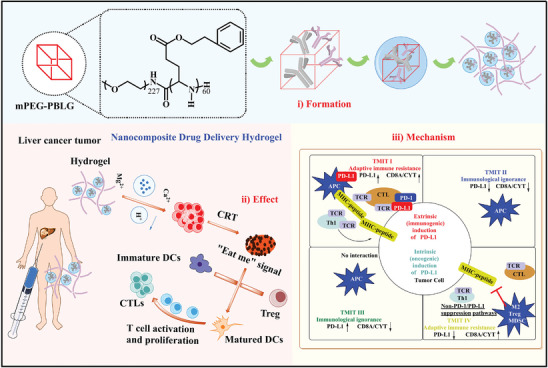
Schematic illustration of the synthetic process of nanocomposite hydrogel A+T@MgCa(CO_3_)_2_@fibrin and treatment mechanism against HCC tumor xenograft on different pathways.

In this investigation, a block copolymer comprising PEG and polyglutamic acid was synthesized and utilized to encapsulate two monoclonal antibody medications, as depicted in (**Scheme** **1**).^[^ ^24^ ^]^ Utilizing methoxy polyethylene glycol with a molecular weight of 5000 as a starting material, the synthesis of block copolymers through the precipitation polymerization method involves the formation of Schiff base. Upon reaching a critical micelle concentration in an aqueous solution, the amphiphilic block copolymer exhibits self‐assembly properties, resulting in the formation of a nano‐scale core‐shell structure. The primary objective of the blocks is to constitute the outer shell of the polymeric micelle, while the hydrophobic blocks contribute to the formation of the hydrophobic core, which can be utilized for the encapsulation of hydrophobic drugs. Despite the satisfactory adsorption capabilities of the block copolymer, its stability remains inadequate. The magnesium calcium carbonate nanoparticles loaded with the monoclonal antibody drug were prepared using the chemical precipitation method, resulting in enhanced stability. Carbonate ions, an indispensable component for human physiology, can also generate carbon dioxide and be eliminated through pulmonary excretion. This drug delivery system exhibits favorable characteristics. Lastly, the formation of a fibrin hydrogel is achieved through the reaction between fibrinogen and thrombin. Overall, the synthesized nanostructured fibrin hydrogel offers significant advantage such as low toxicity, minimal rejection, and moderate viscosity. It can also enrich the loaded drugs in the tumor site, making this nanocomposite hydrogel have certain advantages for the postoperative anti‐recurrence treatment of HCC.

## 2 Results and Discussion

### 2.1 Synthesis and Characterization of the PEG‐PBLG Block Copolymers and Nanoparticles Loaded with Two Antibodies in the Basis of Polymer

**Figure 1:**
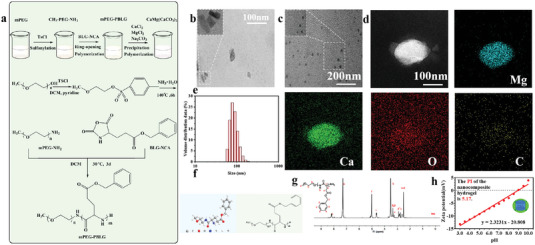
The design, synthesis, and characterizations of nanocomposite particle. a) Schematic route showing the block copolymers. Representative cryo‐scanning electron microscope (SEM) images of b) block copolymers and c) A+T@MgCa(CO_3_)_2_ nanoparticles. Scale bars, 100∼200 nm. Experiments were repeated three times. d) Representative scanning TEM images of A+T@MgCa(CO_3_)_2_ nanoparticles showing the calcium (green), oxygen (red) and carbon(yellow) and Magnesium (blue) (Scale bar: 100 nm). Experiments were repeated three times. e) Average hydrodynamic size of A+T@MgCa(CO_3_)_2_ nanoparticles determined by dynamic light scattering. f) The structural formula of block copolymers. g) ^1^H NMR spectra of block copolymers. h) The zeta potential of nanohydrogel.

It has been found that poly(ethylene glycol)‐polypeptide block copolymers can be used as self‐assembled^[^ ^25^, ^26^, ^27^ ^]^ and nanobiotechnology^[^ ^28^, ^29^, ^30^ ^]^ structures, as well as in various biomedical applications, including drug delivery.^[^ ^31^, ^32^, ^33^, ^34^, ^35^, ^36^ ^]^ It is possible to extensively utilize amphiphilic block copolymers, which consist of PEG and poly(γ‐benzyl‐L‐glutamate) (PBLG), as depots in drug delivery or scaffolds in tissue engineering. In order to prove our hypothesis, we utilized PEG as a functional component in the design of a multivalent host compound. This choice was made because PEG has excellent biocompatibility and a strong attraction to a wide range of guest molecules. Using the Fuchs‐Farthing technique and triphosgene, we initially synthesized the *N*‐carboxy anhydride of *β*‐benzyl‐L‐glutamate. Next, we initiated the NH_2_ amino group of CH_3_O‐PEG‐NH_2_ to polymerize the *N*‐carboxy anhydride of *β*‐benzyl‐L‐glutamate in *N*,*N*‐dimethylformamide, resulting in the synthesis of poly(ethyleneglycol)‐block‐poly(γ‐benzyl‐L‐glutamate) (PEG‐b‐PBLG) block copolymers (BCPs). Experimental details can be found in the Experimental Section. The obtained compound BCPs was characterized by ^1^H NMR. The BCPs was first synthesized as shown (**Figure** **1**a). BCPs was collected and dissolved in an aqueous solution after ultrasonic dispersion and TEM images were taken (Figure 1b). The structural formula and ^1^H NMR spectra of block copolymers were presented (Figure 1f,g).

The compound BCPs synthesized by the above steps is used for subsequent synthesis of block copolymers‐based nanoparticles and preparation of drug loading and hydrogel dispersion. Due to the unique nature of the drug loading characteristics of block copolymer formed by PEG and PBLG, they were utilized in subsequent investigations. As expected, both monoclonal antibody drugs contain cysteine residue domains that can be easily loaded in the presence of PEGylated block copolymers. However, we have observed that the binding affinity is weak, and the drug loading gradually decreases within 24 h. Therefore, we have used this as a crystal nucleus to form MgCa(CO_3_)_2_ nanoparticles through recrystallization, which will enhance the stability of drug loading. Magnesium calcium carbonate is slowly added by a solution of calcium chloride, magnesium chloride, and sodium carbonate to prepare BCPs‐based nanoparticles. Consequently, we acquired uniform A+T@MgCa(CO_3_)_2_ nanoparticles measuring ≈100 nm in diameter. Additionally, a PEG shell was applied to prevent any additional aggregation or agglomeration (Figure 1e). The well‐defined core–shell structure morphology of the A+T@MgCa(CO_3_)_2_ was clearly observed under transmission electron microscopy (TEM) (Figure 1c). The images for elemental mapping showed that Mg^2+^ and Ca^2+^ were evenly distributed in the nanoparticles of MgCa(CO_3_)_2_ (Figure 1d). To examine the alteration in surface potential, the zeta potential of nanocomposite hydrogel was assessed after each synthesis step (Figure 1h). Thus, nanoparticles loaded with the antibody drug A+T are controllable and prepared for subsequent dispersion in hydrogels.

### 2.2 Engineering and Characterization of Crosslinked Fibrin Hydrogel

Nanocomposite hydrogels are scaffolds with 3D structures and can achieve a variety of antitumor properties by incorporating multifunctional nanomaterials.^[^ ^14^, ^37^ ^]^ We then designed the crosslinked fibrin hydrogel to examine whether hydrogel based on biologically delivered A+T@MgCa(CO_3_)_2_@fibrin can protect the tumor from recurrence and metastasis. Fibrin gel was quickly formed by simultaneously spraying and mixing equal volumes of solutions containing either fibrinogen with size‐optimized A+T nanoparticles or thrombin. By enhancing the previous procedure, A+T@MgCa(CO_3_)_2_ nanoparticles were synthesized through the precipitation of Mg^2+^, Ca^2+^, and CO_3_^2−^ in a solution comprising BCPs block copolymers, exhibiting a loading capacity of 5% and an encapsulation efficiency of 50%. To modulate the acidity of the tumor environment, biocompatible calcium carbonate nanoparticles are added to the fibrin gel, acting as a reservoir for immunomodulatory therapeutics and a scavenger of protons. The fibrin hydrogel showed a favorable strain capacity, and storage modulus, which could be well applied to the biological body (Figure S3a–d, Supporting Information). In the acidic buffer (Figure S4a,b, Supporting information), the MgCa(CO_3_)_2_ nanoparticles reacted with H^+^ and caused the dissolution and release of the encapsulated aPD‐L1.The hydrogel effectively trapped A+T@MgCa(CO_3_)_2_ nanoparticles, enabling a controlled and gradual liberation of aPD‐L1. In the meantime, the pH values of the acidic solution were increased by MgCa(CO_3_)_2_ nanoparticles acting as a proton remover, leading to the enhancement of different biological characteristics of the drug gel (Figure S5a–d, Supporting information). Under acidic conditions in the TME, the MgCa(CO_3_)_2_ matrix was disintegrated to release the antibody, Mg ^2+^ and Ca^2+^, which promoted T cell activation and enhanced the cancer immunotherapy.

**Figure 2:**
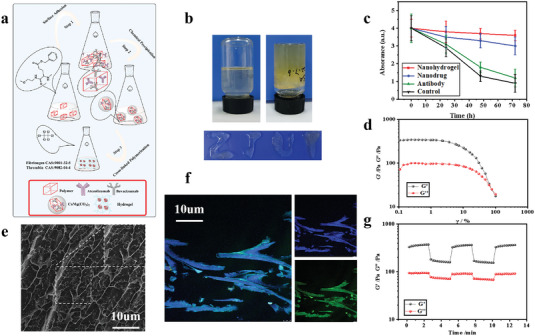
The design, synthesis, and characterizations of nanocomposite hydrogel. a) Schematic illustrating A+T@MgCa(CO_3_)_2_@fibrin synthesis. b) The image of before gelling and after gelling. c) Quantification of the in vivo retention profile of aVEGF–Cy5.5. FL, fluorescence; a.u., arbitrary unit. Data are presented as mean ± s.e.m. (*n* =  3). d) The G′ and G″ in continuous step strain measurements (cycles of 1% and 1000% of strain at 4 min for each cycle). e) Representative cryo‐scanning electron microscope (SEM) images of fibrin gel loaded with A+T@MgCa(CO_3_)_2_ nanoparticles. Scale bars, 10 µm. Experiments were repeated three times. f) Representative fluorescent images of a cryosection of fibrin gel, in which fibrinogen was dispersed with (FITC)‐labeled aVEGF and Cy5.5‐labeled aPD‐L1. Scale bar, 10 µm. Experiments were repeated three times. g) Strain sweep of Gel. The assay was performed at a constant frequency of 1 Hz and 25°C. Statistical significance was calculated via one‐way analysis of variance (ANOVA) with a Tukey post‐hoc test. *P< 0.05.

A rheology test (**Figure** **2**a) confirmed the rapid formation of fibrin gel through the simultaneous spraying and mixing of equal‐volumes of solutions containing either fibrinogen with size‐optimized A+T@MgCa(CO_3_)_2_ nanoparticles or thrombin. Investigation was conducted on the impact of various constituents and pH levels of nano‐components on the formation of gel (see Figure S1a,b, Supporting information). The presence of calcium ions (Ca^2+^) and magnesium ions (Mg^2+^) in the solution promotes the formation of fibrin gel by interacting with MgCa(CO_3_)_2_ nanoparticles. We then continued testing the zeta potential test and shear strength‐strain curves of drug‐loaded nanocomposite hydrogel (Figure S1c,d, Supporting information). The condensed hydrogel has a good solid shape and can be molded into different shapes (Figure 2b). Gels applied to biological wounds have the best retention effects compared to monotherapies and nanoparticles alone (Figure 2c). Combining the two solutions led to a swift rise in the elastic modulus (G′) (Figure 2d; Figure S1d, Supporting information).

Over the entire frequency range tested, the adhesive hydrogel demonstrated viscoelastic properties, with a greater storage modulus (G') than loss modulus (G″), indicating gel‐like behavior (Figure 2g). The nanocomposite hydrogel also exhibited a moderate complex viscosity (η*), suggesting its suitability for application during surgery. The swelling behavior of the nanocomposite hydrogel revealed its ability to absorb a large amount of water, which is essential for drug loading and release. The degradation rate of the nanocomposite hydrogel was found to be moderate, ensuring its long‐term stability and sustained drug release.

Cryo‐SEM imaging (Figure 2e) was utilized to examine the structure of the fibrin gel incorporating A+T@MgCa(CO_3_)_2_ nanoparticles. In order to provide more evidence of the dispersion of A+T@ MgCa(CO_3_)_2_ nanoparticles within the hydrogel, the hydrogel formation involved the use of fluorescein (FITC)‐labeled aVEGF and Cy5.5‐labeled aPD‐L1. Confocal imaging showed that the fibrin displayed a consistent distribution pattern in the gel, with the Cy5.5 and FITC signals. These signals were found to be co‐localized with MgCa(CO_3_)_2_, providing additional evidence of the encapsulation of aPD‐L1 and aVEGF within the MgCa(CO_3_)_2_ nanoparticles (Figure 2f).

### 2.3 In Vivo Evaluation of the Hydrogel Crosslinked with Nanoparticles

**Figure 3:**
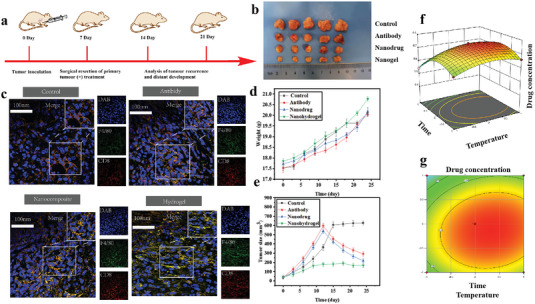
The effection of the in situ formed immunotherapeutic fibrin gel after HCC tumor resection. a) Schematic showing the in situ sprayed bio‐responsive fibrin gel containing A+T@MgCa(CO_3_)_2_ nanoparticles within the post‐surgery tumor bed. b) Digital photographs of the dissected tumors of each group (Scale bar: 1 cm). c) CLSM analysis of tumor uptakes of different agents marked by DAPI, F4/80 and CD8 (Scale bar = 50 µm). d) Time‐dependent body‐weight curves after different treatments. e) Time‐dependent tumor‐growth curves of nude mice (*n* = 5, mean ± s.d.) after different treatments, including control, free A+T, A+T@MgCa(CO_3_)_2_ and A+T@MgCa(CO_3_)_2_ hydrogel. 3D surface plots f) THMs removal g) NOM removal.

In order to assess the in vivo release characteristics, aPD‐L1 and aVEGF were either distributed in phosphate buffered saline (PBS), enclosed within nanoparticles of MgCa(CO_3_)_2_, or incorporated into MgCa(CO_3_)_2_@fibrin gel, and subsequently administered via injection or spraying into the tumor resection cavity. Following optimization, we enhanced the release kinetics of this drug‐loaded nanoparticle‐crosslinked hydrogel by adjusting the releasing time, releasing temperature, and drug concentration. This was done to maximize therapeutic effects and minimize toxicities (**Figure** **3**f,g). Then, the effectiveness of inhibiting therapeutic tumors was assessed in an orthotopic HCC tumor model, using a synergistic approach that directly stimulates immunity through an anti‐PD‐L1 antibody and indirectly targets anti‐angiogenesis with an anti‐VEGF antibody. To create a mouse model of transplanted HCC, C57BL/6 mice were implanted with Hepa1‐6 tumor tissue blocks in their livers. Figure 3a displayed the treatment and monitoring protocol. The mice were randomly assigned to four groups and received injections of different treatment mixtures, including PBS, A+T antibody, A+T loaded with MgCa(CO_3_)_2_, and A+T loaded with MgCa(CO_3_)_2_ encapsulated in fibrin hydrogel. The injections were administered directly at the site in a volume of 100 µL. The dosage of A and T was 10 and 0.5 mg kg^−1^, respectively. Over the course of 14 days, there was no significant decrease in the weight of the animals (Figure 3d). On the 24th day, mice carrying tumors were euthanized, and their tumor tissues were excised, weighed, and photographed. After two weeks of observation of tumor growth, we found that A+T@MgCa(CO_3_)_2_@fibrin can treat HCC with maximum inhibition compared with untreated group (Figure 3e). According to Figure 3b, the weight of orthotopic liver tumors removed on the 24th day indicates that the local tumor load in the combination treatment group was only 23.98 ± 2.47 mg, demonstrating a significant reduction of 95.1% and 94.1% compared to PBS (486.4 ± 129.3 mg) and the A+T group (415.1 ± 73.8 mg), respectively. Large tumors were observed in both the control and A+T antibody groups, as depicted in Figure 3b. However, the tumor size was notably decreased in the A+T@MgCa(CO_3_)_2_ or A+T@MgCa(CO_3_)_2_@fibrin group.

The nanocomposite hydrogel was designed with the fibrin and nanoparticles loading with immune antibodies together. Immune response was induced by stimulating immunity through anti‐PD‐L1 antibody directly and anti‐VEGF antibody for anti‐angiogenesis when the nanocomposite hydrogel was sprayed at the surgical site. The hypoxic tumor microenvironment causes cancer cells to undergo glycolytic metabolism, resulting in the generation of lactate and H^+^ (pH 6.5–6.8). These compounds negatively affect immune cell activities. Inflamed and injured tissues also exhibit local acidification with a pH range of 6.0–7.0. We investigated the immune effects caused by the dispersion of the following four groups within the tumor resection cavity, taking into account the capacity of MgCa(CO_3_)_2_ to remove H^+^ ions. To gain insight into the immune status of the tumors, we conducted immunofluorescence experiments on the tumor tissues from each of the four groups. In Figure 3c, we noticed a decrease in M2‐like tumor‐associated macrophages (CD8F4/80+) and a rise in M1‐like tumor‐associated macrophages (CD8F4/80+). The polarization of macrophages toward the M1 phenotype may be attributed to the alteration of the acidity in the tumor microenvironment (TME), which typically promotes the polarization of macrophages toward an M2‐like phenotype. The MgCa(CO_3_)_2_ scavenging of H^+^ within the inflamed tumor resection offers a straightforward method in contrast to small compounds that induce M1‐like macrophage polarization.

### 2.4 Hydrogel Crosslinked with Nanoparticles for Prevention of Surgical Hemorrhage and Recurrence of Liver Cancer

**Figure 4:**
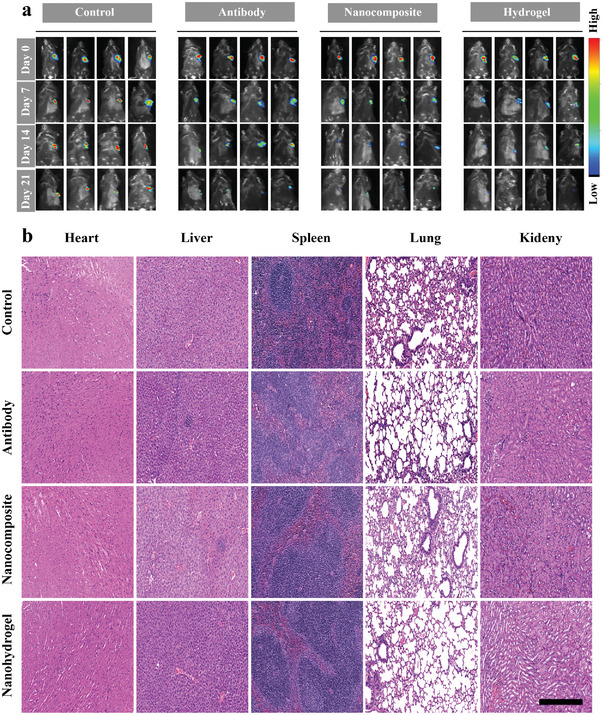
In vivo pharmacokinetic analysis, surveillance, and therapeutic efficacy for HCC tumor‐bearing mice. a) In vivo Fluorescence imaging of mice treated with PBS, A+T, A+T@MgCa(CO_3_)_2_ and A+T@MgCa(CO_3_)_2_@fibrin hydrogel marked by Cy 5.5. b) H&E staining sections of major organs in each group. Sections of the heart, liver, spleen, lung and kidney were stained with H&E to show normal structure and function in each group (scale bar: 50 µm).

To confirm the therapeutic benefits of A+T@MgCa(CO_3_)_2_@fibrin, we employed a tumor resection model that was not fully completed. In the beginning, we established an orthotopic Hepa1‐6 liver cancer model that exhibited a high level of aggressiveness. This model involved the extraction of either half or three‐fourths of the tumor, while preserving a portion of the tumor tissue in its original location to mimic the presence of substantial residual disease after surgical intervention in a clinical setting. In the absence of adjuvant therapy, the tumor consistently exhibits regrowth in all mice due to incomplete surgical excision. As a result, the introduction of supplementary hydrogel therapy in conjunction with checkpoint blockade demonstrates a reduction in tumor regrowth. The fibrin gels formed in the tumor resection cavity contained A+T@MgCa(CO_3_)_2_@fibrin, A+T@MgCa(CO_3_)_2_, A+T or A+T@MgCa(CO_3_)_2_ (1 mg MgCa(CO_3_)_2_ per mouse; 50 µg A+T per mouse) and were applied by spraying. The growth of the tumor was subsequently observed by detecting bioluminescence signals emitted by Hepa1‐6‐FLuc cells (**Figure** **4**a). The treatment with A+T@MgCa(CO_3_)_2_@fibrin did not affect the body weights of 50% of the mice (Figure 3d). Furthermore, examination of the histology of vital organs obtained from mice after 30 days of A+T@MgCa(CO_3_)_2_@fibrin treatment revealed that the localized administration of A+T did not cause any notable adverse reactions in the mice. Following a three‐week period of monitoring tumor development, it was discovered that A+T@MgCa(CO_3_)_2_@fibrin exhibited the highest level of inhibition in treating HCC when compared to the untreated group (Figure 4a). Throughout the 21‐day treatment, the H&E‐stained images of vital organs from all groups (Figure 4b) revealed no apparent signs of toxic side effects, affirming the excellent safety profile of this versatile codelivery nano‐system. Moreover, the utilization of nanocomposite hydrogels in wound management has demonstrated significant efficacy in preventing postoperative bleeding and enhancing the overall safety of surgery procedures. To assess the hemostatic capabilities of the wound dressing, we conducted experiments involving fibrin hydrogel treatment, as depicted in Figure S6a (Supporting information). The in vivo hemostatic performance was evaluated by measuring the amount of blood loss (Figure S6b, Supporting information) and the duration of hemostasis (Figure S6c, Supporting information). These findings unequivocally suggest that the immediate application of nanocomposite hydrogels during surgery effectively mitigates postoperative bleeding and augments the safety of surgical interventions.

### 2.5 RNA‐Seq Data Processing and Analysis of Differentially Expressed Genes and Protein

**Figure 5:**
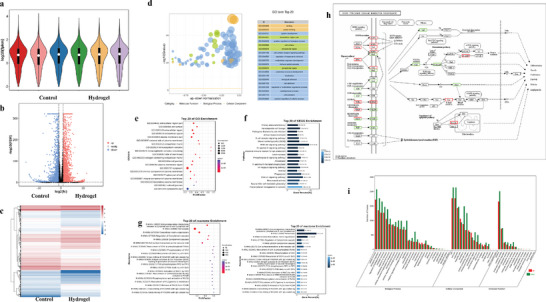
Tumor microenvironment, immune infiltration, and immune checkpoint analyses after nanohydrogel treatment. a) Violin plot analysis comparing the distribution of three different samples in the groups. b) Wilcoxon test Gene difference analysis of nanocomposite hydrogel treatment. c) Heat map of differentially expressed features in nanocomposite hydrogel treatment. d) The Qvalue of top 20 GO term in nanocomposite hydrogel treatment. The top 20 GO enrichment pathways analysis e) and the top 20 KEGG enrichment pathways analysis f) of nanohydrogel treatment. g) The top 20 GO enrichment pathways map of nanocomposite hydrogel treatment. h) The top 20 of reactome enrichment analysis of nanohydrogel treatment. i) The level 2 GO terms of control and nanohydrogel.

In HCC, the benefit of combining nanoparticles with checkpoint inhibitor drugs is still not very clear. RNA sequencing was utilized to examine the kinetics of immune cells in HCC that were subjected to a combination therapy involving anti‐VEGF bevacizumab and the anti‐PD‐L1 atezolizumab encapsulated in nanomaterials. To assess the variations in gene expression levels within remaining tumor cells, a cohort of 12 mice (6 assigned to PBS and 6 to hydrogel) were euthanized subsequent to receiving diverse treatments. The mRNA extracted from their residual tumor tissues was subsequently subjected to analysis. High throughput mRNA sequencing was performed by Novogene to scrutinize these mRNA molecules. Using unsupervised principal component analysis, a total of 13 899 genes that were differentially expressed genes (DEGs) were reduced dimensionally in the entire transcriptome. This reduction included PC1 (41.25%) and PC2 (23.44%). The gene distributions of the PBS and hydrogel groups were easily distinguishable, suggesting that the control group and hydrogel group exhibited distinct gene expression patterns (**Figure** **5**a). In Figure 5b, the volcano plot comparing the control group and hydrogel group reveals that the hydrogel group exhibits 3918 genes that are upregulated and 3703 genes that are downregulated, while the remaining genes are distributed in a non‐significant manner. The study revealed that PBS pretreatment decreased the expression of 962 genes out of the 3918 genes that were up‐regulated by hydrogel treatment (24.55%). Additionally, it up‐regulated 917 genes out of the 3703 genes that were down‐regulated by hydrogel treatment (24.76%). These findings demonstrate that hydrogel pretreatment has the potential to alleviate the abnormal distribution of genes at a pathological level (Figure 5c). Subsequently, we conducted a more in‐depth examination of the pathological process underlying the recurrence and spread of HCC, with particular emphasis on analyzing the DEGs in the PBS group compared to the hydrogel group. These DEGs could be enriched into several KEGG pathways associated with apoptosis, including MAPK, mTOR, PI3K‐Akt, STAT, and TNF signaling pathways (Figure 5d,e), revealing that recurrence and metastasis might be associated with apoptosis. Moreover, the significance value of KEGG enrichment pathways (apoptosis) is 0.01240 (*p, Hydrogel versus PBS). Following the completion of KEGG enrichment analyses, a selection of genes within these pathways was selected to evaluate variations in foldchanges in gene expression across different groups. In the hydrogel group, genes associated with apoptosis, namely Bax, Casp3, and Bid, exhibited up‐regulation in comparison to the PBS group. Conversely, genes linked to anti‐apoptosis, such as Bcl‐2 and Mapk10, demonstrated down‐regulated (Figure 5f,g). Overall, treatment with PBS has the capacity to induce alterations in the distribution of gene expressions associated with programmed cell death. Luckily, the introduction of hydrogel can effectively normalize the distribution of pathological gene alterations, demonstrating that hydrogel treatment can greatly alleviate the fate of apoptotic cell death.

### 2.6 Mechanisms Exploration of the Nanocomposite Hydrogels Inhibiting HCC Recurrence

**Figure 6:**
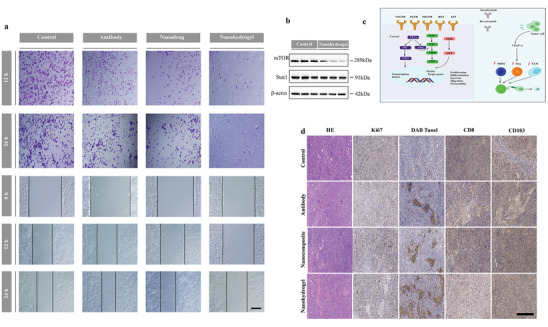
Nanocomposite hydrogel for metastasis and invasion of tumors of hepa1‐6 tumors and mechanism validation. a) Cell scratch tests and transwell assays were carried out to measure proliferation, migration and invasion by Hepa1‐6 cells transfected with indicated vectors. *P<0.05. b) Western blot was performed to detect mTOR expression. c) Schematic showing the in situ sprayed fibrin gel containing A+T@MgCa(CO_3_)_2_ nanoparticles within the post‐surgery tumor bed. d) H&E staining, TUNEL staining, and antigen Ki‐67 immunofluorescence staining for pathological changes in tumor tissues from each group and cellular proliferation (all scale bars: 100 µm).

In the diagram (Figure 5h,i and **Figure** **6**c), we examine and forecast the possible mechanisms of hydrogel therapy for the transportation of medication. To confirm this, we employ western blot assays to authenticate the pathway (Figure 6b). Moreover, the impact of hydrogel therapy on the movement of Hepa 1–6 cells was significantly reversed (P < 0.05, as shown in Figure 6a). In line with the inhibitory regulation of the mTOR and STAT1 signaling pathways in vivo, the hydrogel effectively hindered the activation of these pathways in hepa1‐6 cells (Figure 6b). By activating the inhibitory immune internal environment inside the tumor, the nanocomposite hydrogel can effectively down‐regulate the protein expression level of mTOR related pathway, and then play a related role in inhibiting HCC recurrence.

**Figure 7:**
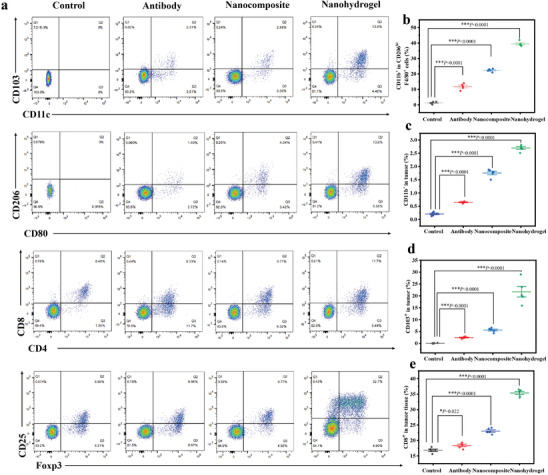
A+T@MgCa(CO_3_)_2_@fibrin hydrogel for triggering antitumor immune response. a) Representative flow cytometric analysis images of four treatment. b) The relative quantification of M1‐like macrophages (CD11bhi) and M2‐like macrophages (CD206hi) gating on F4/80^+^CD11b^+^CD206^+^ cells. Data are presented as mean ± s.e.m. (*n* =  4). c) The quantification results of T cell infiltration within the tumor gating on CD11b^+^ cells in different groups. Data are presented as mean ± s.e.m. (*n* =  4). d) The relative quantification of the CD103^+^ and CD8^+^. e) T cells within tumors following various treatments. Data are presented as mean ± s.e.m. (*n* =  4). Statistical significance was calculated via one‐way ANOVA with a Tukey post‐hoc test. *P< 0.05; **P< 0.01; ***P< 0.001.

Flow cytometry and immunofluorescence staining were employed to analyze the harvested residual tumors 5 days post‐surgery. To uncover the process of combined therapy after different treatments, tumor sections from all mouse groups were collected 24 hours later and stained with hematoxylin and eosin (H&E), TdT‐mediated dUTP Nick‐End Labeling (TUNEL), and Ki‐67 antibody (Figure 6d). Significant apoptosis and necrosis of HCC cells after treatment were indicated by the reduced purple areas (nucleus stained by hematoxylin) in groups (3) and (4) compared to other groups in the H&E staining results. Based on the results of H&E staining, the TUNEL images revealed a significant presence of apoptosis (cells stained brown) in the groups that received synergistic therapy. Notably, the extent of cell apoptosis in group (4) was significantly greater compared to mice administered with free A+T, providing further evidence of the increased effectiveness of the nano‐systems in suppressing HCC. Additionally, the in vivo proliferative activities measured by Ki‐67 antibody staining exhibited stronger suppression (blue‐staining cells) in cell proliferation groups (3) and (4), while all other groups revealed less inhibition of the proliferative activity of cancer cells. In line with the findings from H&E and TUNEL staining, the nanocomposite hydrogel containing A+T@MgCa(CO_3_)_2_@fibrin demonstrated greater suppression of Ki‐67 compared to free A+T, indicating a beneficial inhibitory effect of the drug‐delivery system. Considering the ability of MgCa(CO_3_)_2_ to scavenge H^+^, we examined the immune effects caused by spreading of the four treatments within the tumour resection cavity. We observed an increase of M2‐like TAMs (CD11b^+^CD206^hi^F4/80^+^) (**Figure** **7**b). Remarkably, A+T@MgCa(CO_3_)_2_@fibrin treatment shows an increase in CD11b^+^ dendritic cells, and these cells showed expression of CD8, and CD103, denoting their maturation status (Figure 7c–e). Flow cytometry (Figure 7a; Figure S2, Supporting information) revealed a correlation between the level of MgCa(CO_3_)_2_ nanoparticles and macrophage polarization. This suggests that nanocomposite hydrogels may have the opportunity to reverse the inhibitory tumor microenvironment of HCC.

## 3 Conclusion

In summary, we have successfully developed a nanocomposite hydrogel drug delivery system capable of facilitating the administration of two distinct antibody drugs through different delivery methods. Specifically, our approach involves the direct application of a therapeutic gel to the surgical site following cancer removal, thereby counteracting the immunosuppressive tumor microenvironment and eliciting systemic immune responses. Consequently, this innovative strategy effectively mitigates the risk of local tumor recurrence and metastasis. The inclusion of MgCa(CO_3_)_2_ nanoparticles within a gel matrix has the potential to effectively administer therapeutics in a controlled manner, while also regulating the acidic and inflamed conditions present during tumor resection. This is achieved through the scavenging of H^+^ ions, which subsequently enhances the immune response against tumors. Additionally, the local release of aPD‐L1 by the MgCa(CO_3_)_2_ nanoparticles inhibits the “do not consume me” signal associated with malignant cells, thereby facilitating the elimination of cancerous cells by macrophages. The blockade of PD‐L1 signaling pathway additionally stimulated the immune‐mediated eradication of malignant cells through enhanced presentation of tumor‐specific antigens by macrophages and dendritic cells. These findings substantiate the potential clinical implementation of this approach post‐tumor resection. To accomplish this objective, forthcoming evaluations in comprehensive animal models are expected to optimize drugs dosage, particle quantity, and treatment frequency.

## 4 Experimental Section

### Materials, Cell Lines, and Animals

All chemicals were purchased from Sigma Aldrich and used without any purification. Murine thrombin and fibrinogen were purchased from Molecular Innovations. Bevacizumab (anti‐VEGF, Avastin) and Durvalumab (anti‐PD‐L1) were purchased from Glpbio (cat. no. 216974‐75‐3; cat. no. 1428935‐60‐7). Programmed Cell Death Protein 1 Ligand 1 (PDL1) and Vascular Endothelial Growth Factor (VEGF) enzyme‐linked immunosorbent assay (ELISA) kits were purchased from j&l Biological Industrial Co., Ltd. (Shanghai, China). Hepa1‐6 cell line was obtained from cell bank of Chinese Academy of Sciences. Cells were cultured in Dulbecco's modified Eagle medium (Gibco, Invitrogen) containing 10% fetal bovine serum (Invitrogen) and 100Uml^−1^ penicillin (Invitrogen) in an incubator at 37°C in 5% CO_2_. Female C57BL/6 mice (6–10 weeks) were purchased from the Jackson Lab. All mouse studies were carried out following protocols approved by the animal ethics committee of the Eastern Hepatobiliary Surgery Hospital of Naval Medical University and complied with all relevant ethical regulations (EHBHDW‐2022112).

### Preparation and Characterization of PEG‐b‐P(Glu)

Benzyl glutamic acid and ethyl acetate are mixed in a three‐nozzle flask and heated to reflux, and then the triphosgene is dissolved in the ethyl acetate. The ester solution is slowly added to the three‐necked flask and the heated reflux continues until it is clear. After the reaction is complete, the reaction flask is cooled quickly. Then wash with cold saturated sodium bicarbonate solution and saturated salt water. The upper organic phase is transferred to a single‐necked flask and dried with anhydrous magnesium sulfate. After filtration, the filtrate was concentrated by rotary evaporation. The petroleum ether was frozen to accelerate the precipitation, returned to room temperature, and the crude product was filtered. The crude product was recrystallized with ethyl acetate and petroleum ether to obtain white flaky crystals. The polyethylene glycol‐polybenzyl glutamate (BLG‐NCA) was synthesized subsequently. mPEG10k‐NH_2_ was added to the reaction bottle, a vacuum was heated to remove the water, and freshly distilled bismuth was added under nitrogen protection. Then mPEG10k‐NH_2_ was dissolved in chloromethane; Add anhydrous DMF dissolved BLG‐NCA. Then freshly distilled chloroform was added and reacted at 35 °C for 48 h, and the reaction solution was slowly added to a large amount of water to precipitate. Centrifuge with ethanol, then wash anhydrous ethanol and anhydrous ether and vacuum dry.

### Preparation and Characterization of A+T@MgCa(CO_3_)_2_

A+T@MgCa(CO_3_)_2_ nanoparticles were prepared by chemical precipitation. Typically, 1 mL of Tris‐HCl buffer (1 mm, pH 7.6) containing 100 mm MgCl_2_ and 100 mm CaCl_2_ was mixed with 1 mL HEPES saline buffer (50 mm, pH 7.1, NaCl 140 mm) containing 100 µg Atezoliumab (A) and Bevacizumab(T), 10 mg PEG‐b‐P(Glu) block copolymers (synthesized as previously described) and10mM Na_2_CO_3_. The mixture was stirred for 12 h at 4°C. Excess ions, copolymers and antibodies were removed by centrifugation at 14 800 r.p.m. for 5 min. The size distribution was measured by dynamic light scattering and morphology was evaluated by TEM (JEOL 2000FX). The elemental analysis of A+T@MgCa(CO_3_)_2_ (A+T was chelated gadolinium as previously described) was characterized using analytical TEM (Titan). The amount of aPDL‐1 and aVEGF encapsulated in MgCa(CO_3_)_2_ was measured by enzyme‐linked immunosorbent assay (ELISA). The encapsulation efficiency (EE) and loading capacity (LC) of MgCa(CO_3_)_2_ nanoparticles were calculated using the following formula: EE = (A−B)/A and LC = (A−B)/C, where A is the feed amount of antibody, B is the free non‐entrapped antibody and C is the total weight of particles. The particle size, polydispersity index (PDI), and zeta potential of nanocomposite hydrogels were measured by dynamic light scattering (Malvern Instruments, Malvern).

### Formation and Characterization of A+T@MgCa(CO_3_)_2_@fibrin

Fibrin gels were obtained by spraying equal volumes of fibrinogen (10mg mL^−1^) containing A+T@MgCa(CO_3_)_2_ and thrombin (10NIHUml^−1^). For the control group without MgCa(CO_3_)_2_ nanoparticles, CaCl_2_ and MgCl_2_ was incorporated with the same amount of free Ca^2+^ and Mg^2+^ compared with the solution with MgCa(CO_3_)_2_ nanoparticles. The morphology of A+T@MgCa(CO_3_)_2_@fibrin was characterized by cryo‐SEM (JEOL 7600F, Gatan Alto). Encapsulation of A+T@MgCa(CO_3_)_2_ in fibrin was further characterized by a confocal microscope (Zeiss LSM 710). Dynamic rheological tests were performed on hydrogels using a HAAKE Mars 60 Rheometer (MARS, Germany).

### Release of aVEGF and aPDL1 In Vitro

The release of aVEGF and aPDL1 was studied at 37°C in PBS at various pH values (pH 6.5 and 7.4). Released aVEGF and aPDL1 were measured using ELISA kits.

### Release of aVEGF and aPDL1 In Vivo

To study the in vivo release of aPDL1 and aVEGF, free A+T or A+T@MgCa(CO_3_)_2_@fibrin dispersed in PBS was injected at the tumor resection site. For gel administration, equal volumes of fibrinogen solution (10mg mL^−1^) containing A+T@MgCa(CO_3_)_2_ with the same dose of aPDL1 and aVEGF (50 µg per mouse) and thrombin solution (10NIHUmL^−1^) were sprayed at the tumor resection site. Fluorescence imaging was monitored by an IVIS Spectrum imaging system (Perkin Elmer). To investigate whether the antibody was released from the nanoparticles or still encapsulated in the nanoparticles, intratumoral antibody release behavior was studied using FITC‐labelled MgCa(CO_3_)_2_ nanoparticles. Tumors were collected after different treatments, and the frozen tumor sections were stained with DAPI.

### In Vivo Tumor Models and Treatment

To study the therapeutic effects of A+T@MgCa(CO_3_)_2_@fibrin, 1×10^6^ Hepa 1–6 cells were transplanted into the right flanks of mice. Seven days later, mice were randomly divided into five groups (*n* = 6–8) and tumors were resected leaving ≈1% residual tumor to mimic residual microtumors after surgery. Briefly, mice were anesthetized in an induction chamber using isoflurane (up to 5% for induction; 1–3% for maintenance), and anesthesia was maintained via a nose cone. Sterile instruments were used to remove ≈99% of the tumor. Immediately after surgery, fibrin gels with different formulations, including A+T@MgCa(CO_3_)_2_@fibrin, A+T@MgCa(CO_3_)_2_, A+T with calculated dosages of antibody, were sprayed on the surgical tumor bed by a dual‐cartridge sprayer. The wound was then closed by an Autoclip wound clip system. For the distant tumor model, 7 days after 1×10^6^ Hepa 1–6 were transplanted into the right flank of mice, a second tumor as the distant tumor (1×10^6^ Hepa 1–6) was inoculated into the left flank of each mouse. Three days later, tumors in the right flank were partially resected and the immune therapeutic gel was sprayed on the surgical tumor bed. The tumor size was measured and calculated according to the following formula: width^2^×length×0.5. The tumor was also observed using an in vivo bioluminescence imaging system. Ten minutes after intraperitoneal injection of d‐luciferin (Thermo Scientific Pierce) in DPBS (15mg mL^−1^) into each mouse at a dose of 10 µL g^−1^, mice were imaged using an IVIS Spectrum Imaging System (Perkin Elmer) for 5 min. Regions of interest were quantified as the average radiance (photons s^−1^ cm^−2^ sr^−1^) using Living Image software. Animals were euthanized when showing signs of imperfect health or when the size of their tumors exceeded 1.5 cm^3^.

### Flow Cytometry

Tumors collected from mice were divided into small pieces and homogenized in cold staining buffer to form single‐cell suspensions in the presence of digestive enzymes. Cells were stained with fluorescence‐labeled antibodies CD45 (Biolegend, cat. no. 103 108, clone 30‐F11), CD11b (Biolegend, cat. no. 101 208, clone M1/70), CD206 (Biolegend, cat. no. 141 716, clone C068C2), F4/80 (Biolegend, cat. no. 123 116, clone BM8), CD80 (Biolegend, cat. no. 104 722, clone 16‐10A1), CD3 (Biolegend, cat. no. 100 204, clone 17A2), CD4 (Biolegend, cat. no. 100 412, clone GK1.5), CD8 (Biolegend, cat. no. 140 408, clone 53–5.8), Foxp3 (Biolegend, cat. no. 126 404, clone MF‐14), CD11c (Biolegend, cat. no. 117 310, clone N418), CD86 (Biolegend, cat. no. 105 008, clone GL‐1) and CD103 (Biolegend, cat. no. 121 406, clone 2E7) following the manufacturer's instructions. All antibodies were diluted 200 times. The stained cells were measured on a CytoFLEX flow cytometer (Beckman) and analyzed by FlowJo software (version 10.0.7, TreeStar). The numbers presented in the flow cytometry analysis images are percentage based.

### Confocal Analysis of A+T@MgCa(CO_3_)_2_@fibrin with Different Antibody Distribution In Vitro

In order to measure the retention of different antibody A and T in the A+T@MgCa(CO_3_)_2_@fibrin, A and T antibody was labeled with FITC and CF350 when nanocomposite hydrogel was prepared. A Zeiss confocal fluorescence microscope was used to take fluorescent images after the nanocomposite hydrogel were labeled. Zeiss image processing software was used to analyze the intensity value of the fluorescence.

### Flow Cytometry and Analysis

The tumors were removed from the armpit of the mice after treatment, then cut into small pieces and homogenized on ice. The single‐cell suspension was then prepared with trypsin for detection. When selecting fluorescent‐labeled antibodies, care should be taken to select a combination of different fluorescent channels. The main antibodies involved included anti‐CD4, CD8, Foxp3, IL‐2, TNF‐α, CD103, CD11b, F4/80, CD206, CD80. All the antibodies were diluted 200 times when detected. Staining cells were sampled using a flow cytometer (Beckman) in Univbio and analyzed using FlowJo software (10.7.1). The cell percentage was displayed after the flow cytometry images analysis.

### Immunofluorescence Staining Images

Tumors were dissected from the treated mice and frozen quickly in the medium with optimal cutting temperature (OCT). The tumor tissue is sliced, fixed on a slide, and stained with specific antibodies for 12 h. The antibodies contained: CD3, CD11b, F4/80, CD8. The fluorescence‐labeled secondary goat anti‐rat antibodies were added for further film. The cell nuclei were stained with DAPI. Finally, the sections were imaged and photographed under a Zeiss fluorescence microscope.

### Immunohistochemical Images

Formalin‐fixed paraffin‐embedded (FFPE) method was used for fixing the HCC tumor tissue samples. Antigen repair is performed by heating. The false positive staining rate was reduced by co‐incubation of blocking solution and tissue sections. After incubation with primary and secondary antibodies, Zeiss fluorescence microscopy was used to collect immunohistochemical images.

### In Vivo A+T@MgCa(CO_3_)_2_@fibrin Combined Treatment

To investigate the therapeutic effect of A+T@MgCa(CO_3_)_2_@fibrin, hepa1‐6 cells with stable luciferase expression were transplanted into the right axilla of mice as mentioned. The treatment pattern was the same as A+T@MgCa(CO_3_)_2_@fibrin, with the addition of positive control with anti‐PD‐L1 and anti‐VEGF alone.

### Western Blotting

Equal amounts of protein (measured using a bicinchoninic acid protein assay kit, BCA) were mixed with an equal volume of 2×Laemmli buffer and boiled at 95°C for 5 min. After gel electrophoresis and protein transformation, anti‐mTOR antibody at a dilution of 1:1000 (Abcam, cat. no. ab 39 393), anti‐STAT1 antibody at a dilution of 1:1000 (Santa Cruz, cat. no. ab 109 320) and anti‐β‐actin antibody (Abcam, cat. no. ab8226) at a 1:5000 dilution was used as primary antibodies. The secondary antibody used for these blots was a goat anti‐mouse antibody (Novus Biologicals, cat. no. NBP1‐75151).

### Statistical Analysis

All data are presented as individual values with(or) the mean ± standard error of the mean (s.e.m.). Univariate analysis of variance (ANOVA) was used for data comparison between multiple groups, and Student's *t*‐test was used for data comparison between two groups. Inter‐group comparison of survival analysis was performed by log‐rank test. All statistical analyses were put into practice in GraphPad Software. *P* < 0.05 was considered statistically significant.

## Conflict of Interest

The authors declare no conflict of interest.

## Supporting information

Supporting Information

## Data Availability

The data that support the findings of this study are available from the corresponding author upon reasonable request.

## References

[1] M. Reig , A. Forner , J. Rimola , J. Ferrer‐Fàbrega , M. Burrel , Á. Garcia‐Criado , R. K. Kelley , P. R. Galle , V. Mazzaferro , R. Salem , B. Sangro , A. G. Singal , A. Vogel , J. Fuster , C. Ayuso , J. Bruix , J. Hepatol. 2022, 76, 681. 34801630 10.1016/j.jhep.2021.11.018 PMC8866082

[2] C. Fitzmaurice , T. F. Akinyemiju , F. H. Al Lami , T. Alam , R. Alizadeh‐Navaei , C. Allen , U. Alsharif , N. Alvis‐Guzman , E. Amini , B. O. Anderson , O. Aremu , A. Artaman , S. W. Asgedom , R. Assadi , T. M. Atey , L. Avila‐Burgos , A. Awasthi , H. O. Ba Saleem , A. Barac , J. R. Bennett , I. M. Bensenor , N. Bhakta , H. Brenner , L. Cahuana‐Hurtado , C. A. Castañeda‐Orjuela , F. Catalá‐López , J. J. Choi , D. J. Christopher , S. C. Chung , M. P. Curado , et al., JAMA Oncol 2018, 4, 1553.29860482

[3] N. Kim , J. Cheng , I. Jung , J. D. Liang , Y. L. Shih , W.‐Y. Huang , T. Kimura , V. H. F. Lee , Z. C. Zeng , R. Zhenggan , C. S. Kay , S. J. Heo , J. Y. Won , J. Seong , J. Hepatol. 2020, 73, 121. 32165253 10.1016/j.jhep.2020.03.005

[4] X. Wang , F. Meng , Y.‐T. Yen , R. Li , B. Liu , Adv. Funct. Mater. 2021, 31, 2004713.

[5] D. M. Smith , J. K. Simon , J. R. Baker Jr , Nat. Rev. Immunol. 2013, 13, 592. 23883969 10.1038/nri3488 PMC7097370

[6] R. S. Riley , C. H. June , R. Langer , M. J. Mitchell , Nat Rev Drug Discov 2019, 18, 175. 30622344 10.1038/s41573-018-0006-z PMC6410566

[7] J. Hong , M. Kang , M. Jung , Y. Y. Lee , Y. Cho , C. Kim , S. Y. Song , C. G. Park , J. Doh , B.‐S. Kim , Adv. Mater. 2021, 33, 2101110. 10.1002/adma.202101110 34235790

[8] N. Gong , N. C. Sheppard , M. M. Billingsley , C. H. June , M. J. Mitchell , Nat. Nanotechnol. 2021, 16, 25. 33437036 10.1038/s41565-020-00822-y

[9] D. J. Irvine , E. L. Dane , Nat. Rev. Immunol. 2020, 20, 321. 32005979 10.1038/s41577-019-0269-6 PMC7536618

[10] J. Bruix , S. L. Chan , P. R. Galle , L. Rimassa , B. Sangro , J. Hepatol. 2021, 75, 960. 34256065 10.1016/j.jhep.2021.07.004

[11] M. Pinter , B. Scheiner , M. Peck‐Radosavljevic , Gut 2021, 70, 204. 32747413 10.1136/gutjnl-2020-321702 PMC7788203

[12] J. Meng , X. Yang , J. Huang , Z. Tuo , Y. Hu , Z. Liao , Y. Tian , S. Deng , Y. Deng , Z. Zhou , J. F. Lovell , H. Jin , Y. Liu , K. Yang , Adv. Sci. 2023, 10, e2300517. 10.1002/advs.202300517 PMC10369279 37132587

[13] X. Liu , Y. Huangfu , J. Wang , P. Kong , W. Tian , P. Liu , C. Fang , S. Li , Y. Nie , Z. Feng , P. Huang , S. Shi , C. Zhang , A. Dong , W. Wang , Adv. Sci. 2023, 10, e2300637. 10.1002/advs.202300637 PMC10401096 37229748

[14] J. Zhang , C. Chen , A. Li , W. Jing , P. Sun , X. Huang , Y. Liu , S. Zhang , W. Du , R. Zhang , Y. Liu , A. Gong , J. Wu , X. Jiang , Nat. Nanotechnol. 2021, 16, 538. 33526838 10.1038/s41565-020-00843-7

[15] A. X. Zhu , A. R. Abbas , M. R. De Galarreta , Y. Guan , S. Lu , H. Koeppen , W. Zhang , C.‐H. Hsu , A. R. He , B.‐Y. Ryoo , T. Yau , A. O. Kaseb , A. M. Burgoyne , F. Dayyani , J. Spahn , W. Verret , R. S. Finn , H. C. Toh , A. Lujambio , Y. Wang , Nat. Med. 2022, 28, 1599. 35739268 10.1038/s41591-022-01868-2

[16] E. Piantanida , G. Alonci , A. Bertucci , L. De Cola , Acc. Chem. Res. 2019, 52, 2101. 31291090 10.1021/acs.accounts.9b00114

[17] J. Conde , N. Oliva , M. Atilano , H. S. Song , N. Artzi , Nat. Mater. 2016, 15, 353. 26641016 10.1038/nmat4497 PMC6594154

[18] J. D. Weaver , D. M. Headen , J. Aquart , C. T. Johnson , L. D. Shea , H. Shirwan , A. J. García , Sci. Adv. 2017, 3, e1700184. 28630926 10.1126/sciadv.1700184 PMC5457148

[19] K. Roshanbinfar , L. Vogt , B. Greber , S. Diecke , A. R. Boccaccini , T. Scheibel , F. B. Engel , Adv. Mater. 2018, 28, 1803951.

[20] Y. Chen , J. Shi , Y. Zhang , J. Miao , Z. Zhao , X. Jin , L. Liu , L. Yu , C. Shen , J. Ding , J. Mater. Chem. B 2020, 8, 980. 31930242 10.1039/c9tb02523e

[21] X. Yang , X. Chen , Y. Wang , G. Xu , L. Yu , J. Ding , Chem. Eng. J. 396, 125320.

[22] J. W. Yang , R. B. Bai , B. H. Chen , Z. G. Suo , Adv. Mater. 2020, 30, 1901693.

[23] J. Chen , D. Wang , L.‐H. Wang , W. Liu , A. Chiu , K. Shariati , Q. Liu , X. Wang , Z. Zhong , J. Webb , R. E. Schwartz , N. Bouklas , M. Ma , Adv. Mater. 2020, 32, 2001628. 10.1002/adma.202001628 PMC7606513 32945035

[24] Q. Chen , C. Wang , X. Zhang , G. Chen , Q. Hu , H. Li , J. Wang , D. Wen , Y. Zhang , Y. Lu , G. Yang , C. Jiang , J. Wang , G. Dotti , Z. Gu , Nat. Nanotechnol. 2019, 14, 89. 30531990 10.1038/s41565-018-0319-4

[25] M. Elsabahy , K. L. Wooley , Chem. Soc. Rev. 2012, 41, 2545. 22334259 10.1039/c2cs15327k PMC3299918

[26] Z. Song , Z. Han , S. Lv , C. Chen , L. Chen , L. Yin , J. Cheng , Chem. Soc. Rev. 2017, 46, 6570. 28944387 10.1039/c7cs00460e

[27] Z. Song , H. Kim , X. Ba , R. Baumgartner , J. S. Lee , H. Tang , C. Leal , J. Cheng , Soft Matter 2015, 11, 4091. 25939493 10.1039/c5sm00820d

[28] Y. Bae , K. Kataoka , Adv. Drug Delivery Rev. 2009, 61, 768. 10.1016/j.addr.2009.04.016 19422866

[29] C. Cai , J. Lin , Y. Lu , Q. Zhang , L. Wang , Chem. Soc. Rev. 2016, 45, 5985. 27722321 10.1039/c6cs00013d

[30] X. Zhou , Z. Li , Adv. Healthcare Mater. 2018, 7, e1800020. 10.1002/adhm.201800020 29869375

[31] S. Deshayes , H. Cabral , T. Ishii , Y. Miura , S. Kobayashi , T. Yamashita , A. Matsumoto , Y. Miyahara , N. Nishiyama , K. Kataoka , J. Am. Chem. Soc. 2013, 135, 15501. 24028269 10.1021/ja406406h

[32] Y. Mochida , H. Cabral , Y. Miura , F. Albertini , S. Fukushima , K. Osada , N. Nishiyama , K. Kataoka , ACS Nano 2014, 8, 6724. 24927216 10.1021/nn500498t

[33] M. A. Quadir , S. W. Morton , Z. J. Deng , K. E. Shopsowitz , R. P. Murphy , T. H. Epps , P. T. Hammond , Mol. Pharmaceutics 2014, 11, 2420. 10.1021/mp500162w PMC4096223 24813025

[34] K. Wang , Y. Liu , C. Li , S.‐X. Cheng , R.‐X. Zhuo , X.‐Z. Zhang , ACS Macro Lett. 2013, 2, 201. 35581882 10.1021/mz300568b

[35] S. Lv , Z. Tang , M. Li , J. Lin , W. Song , H. Liu , Y. Huang , Y. Zhang , X. Chen , Biomaterials 2014, 35, 6118. 24794923 10.1016/j.biomaterials.2014.04.034

[36] S. Lv , Y. Wu , K. Cai , H. He , Y. Li , M. Lan , X. Chen , J. Cheng , L. Yin , J. Am. Chem. Soc. 2018, 140, 1235. 29332390 10.1021/jacs.7b12776

[37] S. Correa , A. K. Grosskopf , H. Lopez Hernandez , D. Chan , A. C. Yu , L. M. Stapleton , E. A. Appel , Chem. Rev. 2021, 121, 11385. 33938724 10.1021/acs.chemrev.0c01177 PMC8461619

