# Supplementary material for: Hydrogel Crosslinked with Nanoparticles for Prevention of Surgical Hemorrhage and Recurrence of Hepatocellular Carcinoma
Source: Adv Sci (Weinh). 2023 Dec 25;11(9):2305508. doi: 10.1002/advs.202305508 (PMC10916646; doi:10.1002/advs.202305508)
Supplement: Supplementary file 1 — Supporting Information [file ADVS-11-2305508-s001.pdf]

ADVANCED SCIENCE
Open Access

Supporting Information

for *Adv. Sci.*, DOI 10.1002/advs.202305508

Hydrogel Crosslinked with Nanoparticles for Prevention of Surgical Hemorrhage and Recurrence of Hepatocellular Carcinoma

Jia-Qi Zhu, Han Wu, Xu Li, Min-Yu Li, Zhen-Li Li, Xin-Fei Xu, Li-Hui Gu, Dong-Xu Yin, Feng Shen, Dong-Sheng Huang and Tian Yang**

Supporting Information

Hydrogel Crosslinked with Nanoparticles for Prevention of Surgical Hemorrhage and Recurrence of Hepatocellular Carcinoma

Jia-Qi Zhu *PhD*[1,2#], Han Wu *MD*[1#], Xu Li *MD*[3#], Min-Yu Li *MD*[4#], Zhen-Li Li *MD*[1], Xin-Fei Xu *MD*[1], Li-Hui Gu *MD*[1], Dong-Xu Yin *MD*[1,6], Feng Shen *MD*[1], Dong-Sheng Huang *MD, PhD*[2,5,6*], Tian Yang *MD*[1,6*]

[1] Department of Hepatobiliary Surgery, Eastern Hepatobiliary Surgery Hospital, Second Military Medical University (Naval Medical University), Shanghai 200438, China

[2] College of Biotechnology and Bioengineering, Zhejiang University of Technology, Hangzhou, Zhejiang 310014, China

[3] Department of Colorectal Surgery, The First Affiliated Hospital of Naval Medical University，Shanghai 200433, China

[4] Department of Special Care Unit, The First Affiliated Hospital of Naval Medical University, Shanghai 200433, China

[5] The Key Laboratory of Tumor Molecular Diagnosis and Individualized Medicine of Zhejiang Province, Zhejiang Provincial People's Hospital (People's Hospital of Hangzhou Medical College), Hangzhou, Zhejiang 310014, China

[6] School of Clinical Medicine, Hangzhou Medical College, Hangzhou, Zhejiang 310014, China

***Correspondence to:** Prof. Tian Yang, MD, Department of Hepatobiliary Surgery, Eastern Hepatobiliary Surgery Hospital, Second Military Medical University (Naval Medical University), No. 225, Changhai Road, Shanghai 200438, China; or School of Clinical Medicine, Hangzhou Medical College, Hangzhou, Zhejiang 310014, China; E-mail: yangtiandfgd@hotmail.com; Or Prof. Dong-Sheng Huang, MD, The Key Laboratory of Tumor Molecular Diagnosis and Individualized Medicine of Zhejiang Province, Zhejiang Provincial People’s Hospital (People’s Hospital of Hangzhou Medical College), Hangzhou, China or School of Clinical Medicine, Hangzhou Medical College, No. 158, Shangtang Road, Hangzhou, Zhejiang 310014, China; E-mail: huangdongshengzj@hotmail.com;

Short Title: Nanocomposite Hemostatic Hydrogels for HCC Anti-recurrence Treatment

Keywords: Adhesive hydrogels, immunotherapy, hepatocellular carcinoma, block copolymer, tumor recurrence

# These authors contribute equally to this work.

Figures: 6

Pages: 7

CONTENTS of FIGURES

Figure S1. The different formations of different hydrogels.

Figure S2. A+T@MgCa(CO_3)$_2$@fibrin hydrogel for triggering antitumor immune response.

Figure S3. Characterization of A+T@MgCa(CO_3)$_2$@fibrin hydrogel.

Figure S4. Residual activity of A+T@MgCa(CO_3)$_2$@fibrin hydrogel.

Figure S5. The characterization of single drug and nanocomposite hydrogel.

Figure S6. Hemostatic performance of the wound dressing in different treatment.

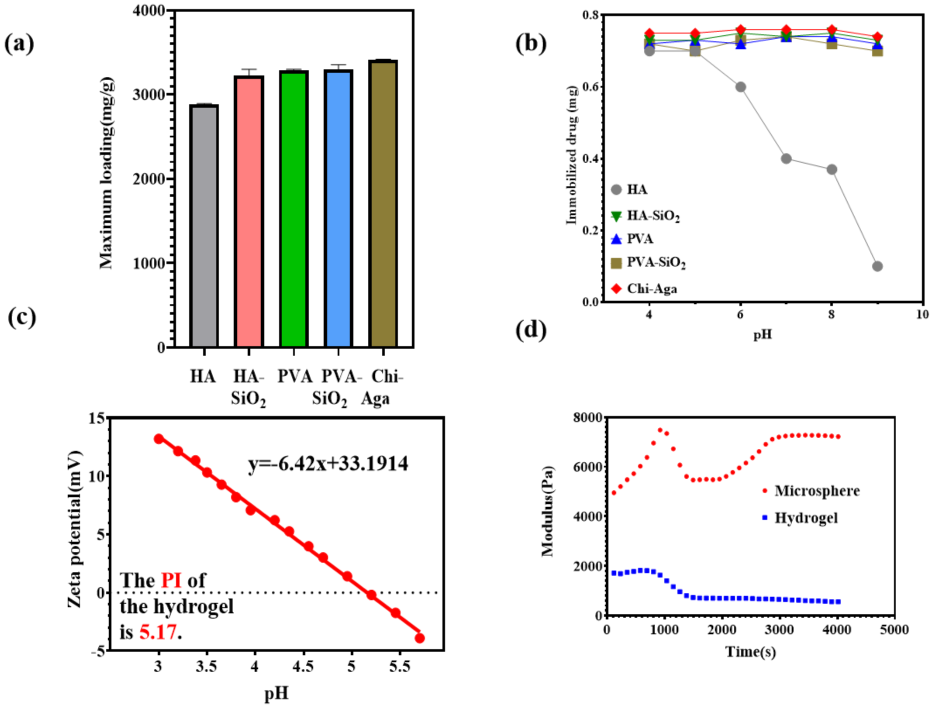

Figure S1. The different formations of different hydrogels. (a) Different materials for nanocomposite hydrogel synthesis; (b) The impact of pH for the synthesize of drug loaded nanocomposite hydrogel; (c)The zeta potential test of drug-loaded nanocomposite hydrogel; (d)Shear strength-strain curves of hydrogel in gel and microsphere shape.

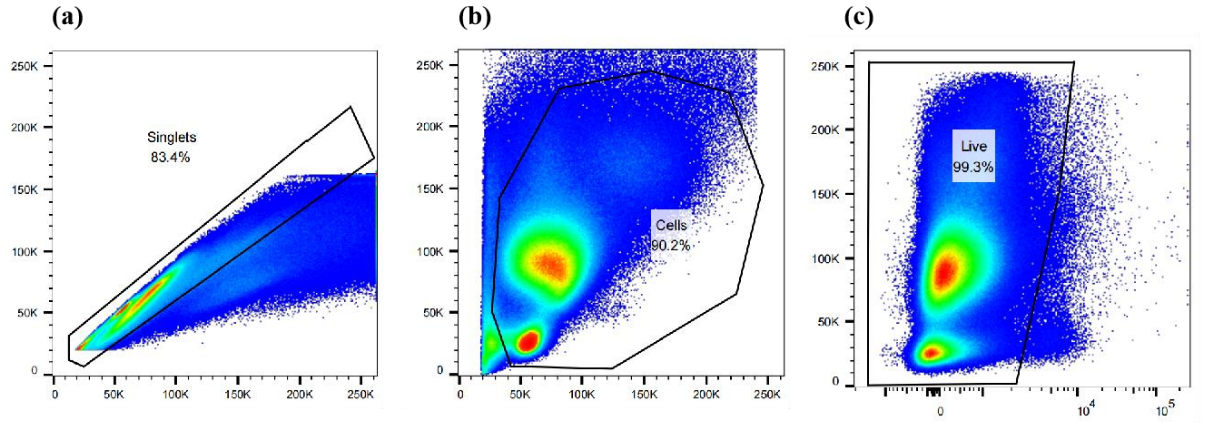

Figure S2. A+T@MgCa$(CO_3)_2$@fibrin hydrogel for triggering antitumor immune response. Representative flow cytometric analysis images (a) and corresponding quantification cell (b). In it, the live part is shown in (c).

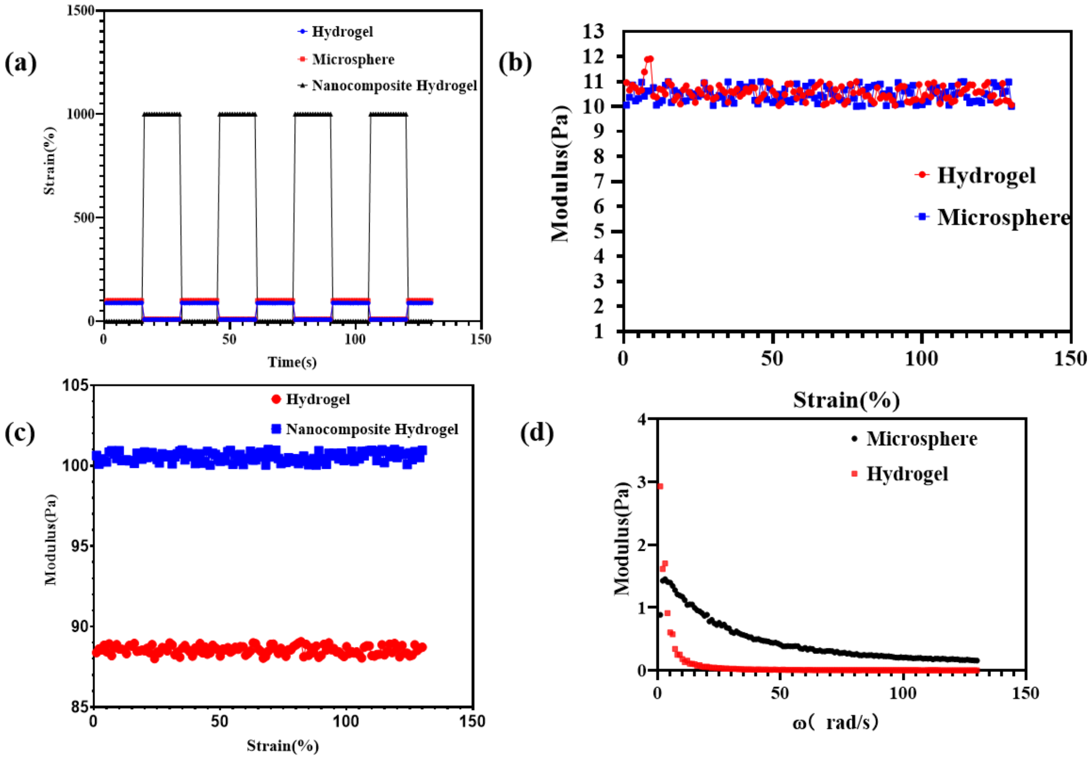

Figure S3. Characterization of A+T@MgCa$(CO_3)_2$@fibrin hydrogel. (a) Strain capacity (b) Storage modulus (c) Loss modulus and (d) Composite viscosity of different hydrogels.

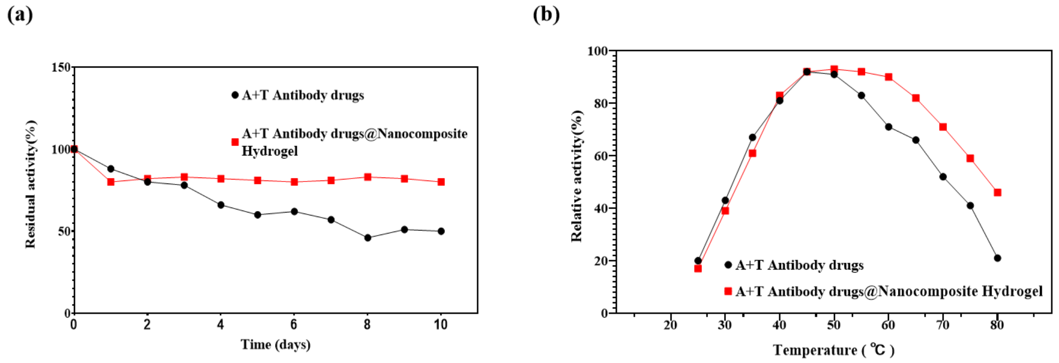

Figure S4. Residual activity of A+T@$MgCa(CO_3)_2$@fibrin hydrogel. (a)The drug release process of single drug and nanocomposite hydrogel; (b)The stability of single drug and nanocomposite hydrogel in different temperatures.

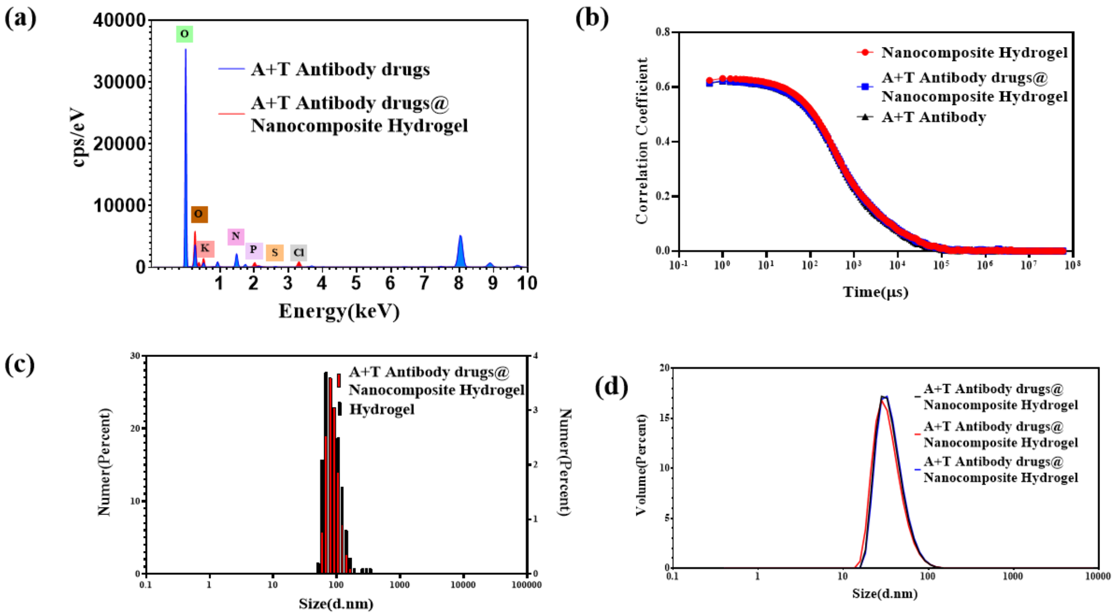

Figure S5. The characterization of single drug and nanocomposite hydrogel. (a) cps curve of single drug and nanocomposite hydrogel; (b) correlation coefficient of hydrogel and nanocomposite hydrogel; The number (c) and volume (d) of drug loaded nanocomposite hydrogel.

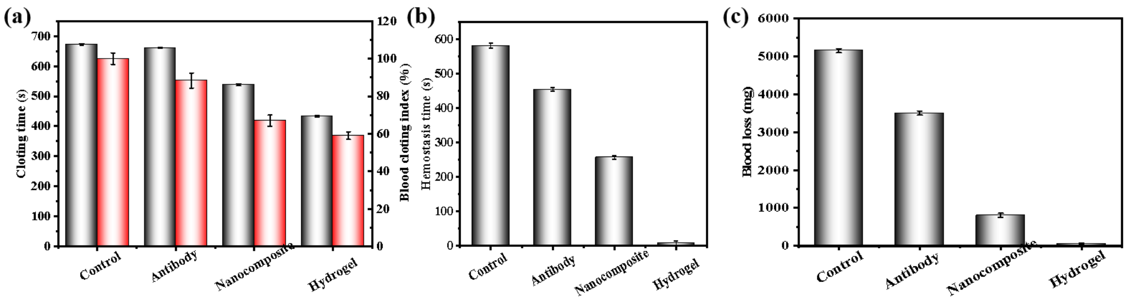

Figure S6. Hemostatic performance of the wound dressing in different treatment. (a) In vitro coagulation time and blood-clotting index (BCI) of different groups. In vivo hemostatic performance was evaluated by recording(b) blood loss and (c) hemostatic time.
